# Validation of the ICEBERG emergency room screening tool for early identification of older patients with geriatric consultation needs

**DOI:** 10.3389/fmed.2023.1240082

**Published:** 2023-09-27

**Authors:** Heike A. Bischoff-Ferrari, Michael Gagesch, Dai-Hua Tsai, Clara Richter, Patricia Lanz, Patrick Sidler, Uenal Can, Dagmar I. Keller, Markus Minder, Bettina von Rickenbach, Ali Yildirim-Aman, Katharina Geiling, Gregor Freystaetter

**Affiliations:** ^1^Center on Aging and Mobility, University Hospital Zurich and University of Zurich, Zurich, Switzerland; ^2^University Clinic for Aging Medicine, City Hospital Zurich, Zurich, Switzerland; ^3^Department of Aging Medicine, University Hospital Zurich, Zurich, Switzerland; ^4^Institute for Emergency Medicine, City Hospital Zurich, Zurich, Switzerland; ^5^Institute for Emergency Medicine, University Hospital Zurich, Zurich, Switzerland; ^6^Center for Aging Medicine and Palliative Care, Hospital Affoltern, Affoltern, Switzerland; ^7^Interdisciplinary Emergency Center, Hospital Affoltern, Affoltern, Switzerland

**Keywords:** geriatrics, older patients, emergency room, screening tool, validation, ICEBERG

## Abstract

**Background:**

The growing number of older and oldest-old patients often present in the emergency room (ER) with undiagnosed geriatric syndromes posing them at high risk for complications in acute care.

**Objective:**

To develop and validate an ER screening tool (ICEBERG) to capture 9 geriatric domains of risk in older patients.

**Design, setting, and participants:**

For construct validity we performed a chart-based study in 129 ER patients age 70 years and older admitted to acute geriatric care (pilot 1). For criterion validity we performed a prospective study in 288 ER patients age 70 years and older admitted to acute care (pilot 2).

**Exposure:**

In both validation steps, the exposure was ICEBERG test performance below and above the median score (10, range 0–30).

**Outcome measures and analysis:**

In pilot 1, we compared the exposure with results of nine tests of the Comprehensive Geriatric Assessment (CGA). In pilot 2, we compared the exposure assessed in the ER to following length of hospital stay (LOS), one-on-one nursing care needs, in-hospital mortality, 30-day re-admission rate, and discharge to a nursing home.

**Main results:**

Mean age was 82.9 years (SD 6.7; n = 129) in pilot 1, and 81.5 years (SD 7.0; n = 288) in pilot 2. In pilot 1, scoring ≥10 was associated with significantly worse performance in 8 of 9 of the individual CGA tests. In pilot 2, scoring ≥10 resulted in longer average LOS (median 7 days, IQR 4, 11 vs. 6 days, IQR 3, 8) and higher nursing care needs (median 1,838 min, IQR 901, 4,267 vs. median 1,393 min, IQR 743, 2,390). Scoring ≥10 also increased the odds of one-on-one nursing care 2.9-fold (OR 2.86, 95%CI 1.17–6.98), and the odds of discharge to a nursing home 3.7-fold (OR 3.70, 95%CI 1.74–7.85). Further, scoring ≥10 was associated with higher in-hospital mortality and re-hospitalization rates, however not reaching statistical significance. Average time to complete the ICEBERG tool was 4.3 min (SD 1.3).

**Conclusion:**

Our validation studies support construct validity of the ICEBERG tool with the CGA, and criterion validity with several clinical indicators in acute care.

## 1. Introduction

In Europe and the US the number of adults age 65 and older is expected to double by 2050 (1). As older adults are more vulnerable to chronic and acute diseases, admission rates to the Emergency Room (ER) and acute care are expected to grow considerably in the coming years (2). Further, older patients often present in the ER with competing geriatric risks including multimorbidity, polypharmacy, and functional impairments, which are independent predictors of acute care outcomes. However, competing geriatric risks are not regularly assessed in the ER setting (3). This is in part explained by time constraints (4). For example, it has been suggested that among patients admitted to the ER with delirium, only one third may be identified by routine clinical observation (5).

A Comprehensive Geriatric Assessment (CGA) is the accepted gold standard to identify risks and impairments in older adult patients admitted to hospital care (6). It provides the basis to develop an optimal management plan to provide integrated care and improved outcomes in this vulnerable patient group (4). However, due to the limited availability of geriatric expertise and time constraints in the busy ER environment, performing a front-door CGA in this scenario often appears not feasible (4).

Consequently, several case finding tools, including the Identification of Seniors At Risk (ISAR) tool, Triage Risk Screening Tool (TRST), Short Emergency Geriatric Assessment (SEGA), Silver code, and Brief Risk Identification for Geriatric Health Tool (BRIGHT) have been proposed to identify older ER patients at-risk for adverse outcomes (7–12). However, so far none of these tools demonstrated high predictive validity with regard to relevant clinical outcomes in acute care (13–15). Furthermore, most of the existing tools only partially reflect the geriatric risk profile (i.e., comorbidities, cognition, nutrition, self-care needs, polypharmacy, mobility, and fall risk) relevant to the evaluation of older patients in the ER setting.

In summary, to the best of our knowledge, a practical and yet comprehensive and reliable tool to identify older adult patients with geriatric syndromes who would benefit from geriatric consultation is timely and missing to date.

Therefore, we set out to develop ICEBERG as a practical screening tool to cover nine geriatric risk dimensions associated with adverse outcomes in acute care, based on the current evidence for improved outcomes (16). And then to validate its construct validity against the CGA and its criterion validity with regard to relevant clinical outcome indicators in acute care.

## 2. Materials and methods

### 2.1. Study design and participants

**Figure 1:**
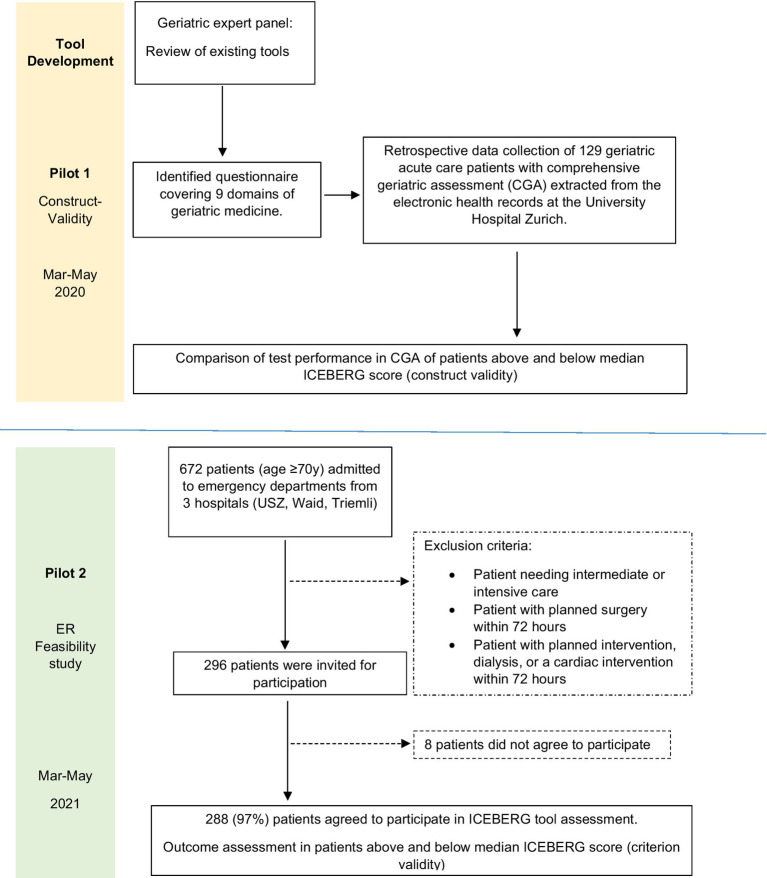
Study schema ICEBERG.

Our study included a development phase and 2 pilot studies on construct validity (Pilot 1) and criterion validity (Pilot 2) for the novel ICEBERG tool (see Figure 1).

#### 2.1.1. ICEBERG tool development

We assembled a clinical expert panel of geriatricians to develop a new screening tool for early identification of geriatric consultation need among older patients admitted to acute care via the ER. First, the expert panel reviewed the available literature to identify screening elements to represent these domains with potential feasibility in the ER setting. This step was coordinated by a Geriatric fellow (Angélique Sadlon, MD). To best complement the existing literature and tools, the expert panel considered pre-existing scoring instruments that had been developed for use in the ER (7–12). The tool was designed to capture nine core geriatric domains of a CGA associated with adverse outcomes in the acute care setting as described in a systematic review and meta-analysis by Häseler-Ouart et al. (16), including 1) social situation, 2) patients’ age, 3) mobility and falls, 4) cognitive impairment and depression, 5) delirium, 6) prior hospitalization, ER or unplanned primary care consultation, 7) number of medications, 8) presence of functional limitations, and 9) malnutrition.

Candidate items and their weighting in the existing literature were evaluated for their relevance to form the nine domain ICEBERG tool. Table 1 shows the overlap of the nine geriatric domains of ICEBERG compared with existing screening ER tools. Table 2 illustrates the nine domains of ICEBERG with a total score ranging from 0 to 30, where higher scores indicate the presence of more geriatric syndromes or impairments. Thus, representing a greater need for geriatric consultation. Overall scores were categorized as either ‘high ICEBERG (≥10)’ or ‘low ICEBERG (<10)’ based on the median total score obtained from both, pilot 1 (construct validity) and pilot 2 (criterion validity). The ICEBERG tool was designed to allow both, self-report by the patients’ or proxy-report by accompanying relatives or care-givers, plus information extracted from the electronic patient record.

**Table 1:** Geriatric domains covered by geriatric screening tools available for use in ER settings.

*Tools*									
	Domain								
	Q1. Social situation	Q2. Patient’s age	Q3. Gait disturbances (Falls)	Q4. Baseline Cognitive function/ Depression	Q5. Delirium	Q6. Past hospitalization/ ER visit	Q7. Polypharmacy (Medication)	Q8. Frailty (Functional limitation)	Q9. Malnutrition
ICEBERG	x	x	x	x	x	x	x	x	x
ISAR	x			x		x	x	x	
TRST	x		x	x		x	x		
SEGA	x	x	x	(x)*	x	x	x	x	x
Silver code		x				x	x	x	
BRIGHT	x		x	x	x			x	

ISAR: Identification of Seniors At Risk (7); TRST: Triage Risk Screening Tool (10); SEGA: Short Emergency Geriatric Assessment (11); Silver Code (8); BRIGHT: Brief Risk Identification for Geriatric Health Tool (9).

* SEGA only covers current cognitive status upon ER admission.

**Table 2:** ICEBERG tool (score range 1 to 30).

ICEBERG questionnaire answers (score)	Score
**Q1: Where does the patient live?** □ Living independently at home without support (0) □ At home with support from family member and/or outside help (eg SPITEX) (2) □ In a nursing home (3)	
**Q2: What is the patient's age?** □ 70-79 years (1) □ ≥ 80 years (2)	
**Q3a: Falls (2+) in the last 12 months?** □ Yes (3) □ No (0) **Q3b: Is a fall the reason for the current ER visit?** □ Yes (1) □ No (0)	
**Q4a: Is there any sign for a cognitive disorder impacting everyday life activities?** □ Yes (3) □ No (0) **Q4b: Has the patient felt down, depressed, or fatigued lately?** □ Yes (2) □ No (0)	
**Q5: Can the patient count the months of the year backwards without ANY ERROR up to and including September?** □ Yes (0) □ No (3)	
**Q6a: ER or emergency visit at GP in past month?** □ Yes (2) □ No (0) **Q6b: Hospitalization in past 6 months?** □ Yes (2) □ No (0)	
**Q7: How many drugs does the patient take regularly?** □ Between 0 and 3 drugs (0) □ Between 4 and 7 drugs (1) □ ≥ 8 drugs (2)	
**Q8a: Does the patient usually need any help with taking showers or bathing?** □ Yes (2) □ No (0) **Q8b: Right now, can the patient transfer from a lying or sitting position to a standing position independently?** □ Yes (0) □ No (2)	
**Q9a: Has the patient lost more than 3 kg weight without trying and/or do the clothes not fit anymore?** □ Yes (2) □ No (0) **Q9b: Has the patient been eating poorly because of decreased appetite?** □ Yes (1) □ No (0)	

#### 2.1.2. Pilot 1 (construct validity)

Data were retrospectively extracted from electronic health records of 129 patients admitted to the Dept. of Aging Medicine at the University Hospital Zurich (USZ) between January and June 2019 via the ER and undergoing a CGA upon admission to the geriatric ward. Performing a CGA is the considered gold standard instrument to comprehensively capture the core geriatric domains of health and functioning among older patients forming the basis of a personalized treatment plan (17). From the standard CGA at the Dept. of Aging Medicine at the USZ, the following components were used for the present study: (1) Barthel Index (range 0–100) (18), (2) self-care index (SPI, range 0–40) (19), (3) Mini Mental State Examination (MMSE, range 0–30) (20), (4) Clock drawing test (range 0–7) (21), (5) Mini Nutritional Assessment (MNA, range 0–30) (22), (6) bilateral Handgrip strength test (23), (7) Short Physical Performance Battery (SPPB, range 0–12) (24), (8) Frailty according to Fried (Frailty-Fried, range 0–5) (25), and (9) Frailty score according to SHARE (Frailty-SHARE, range 0–5) (26). Performing a CGA takes about one hour plus time for documentation. For each included patient, the CGA was performed by a fully-trained geriatric assessment nurse and/or geriatrician. Based on electronic patient data, we also extracted additional information relevant to calculate the ICEBERG score for each of the included patients. The performance in each of the CGA components was compared for ICEBERG score performance below and above the median (≥10 vs. <10 points).

#### 2.1.3. Pilot 2 (feasibility and criterion validity)

For our prospective pilot study, we recruited 288 consecutive patients age 70 years and older admitted to acute care from three ER’s in the City of Zurich, Switzerland (USZ, City Hospital Zurich – Waid, City Hospital Zurich – Triemli) between March and May 2021. Pilot 2 was designed to assess the feasibility of the ICEBERG tool in the ER setting and to test criterion validity of the ICEBERG tool with regard to several clinical outcome indicators in acute care collected prospectively. Patients were excluded in pilot 2 if they did not require inpatient care or if they needed critical care unit admission directly from the ER or immediate surgery or urgent medical intervention (e.g., dialysis or a cardiac intervention) within 72 h upon admission. As shown in Figure 1, 296 patients provided written consent to participate in pilot 2, and only 8 patients declined participation. A total of 288 (97%) patients were included in the final analysis of pilot 2.

To assess the **feasibility** of implementing the ICEBERG tool in the ER setting, willingness to provide consent and the time (minutes) required to complete the questionnaire was recorded. In addition, we documented the source of information (i.e., from electronic health records, patient, or proxy) for each question, and how the patient rated the level of difficulty to respond to each question of the ICEBERG tool.

**Criterion validity** was based on the following prospective outcomes for each enrolled patient: Length of hospital stay (LOS) (days), level of nursing care (minutes), in-hospital mortality (yes/no), need of one-on-one nursing care (i.e., “sitters”) (yes/no), discharge to a nursing home among patients admitted from home (yes/no), and re-hospitalization within 30 days (yes/no).

The Cantonal Ethics Committee of Zurich declared that authorization from the ethics committee was not required as this project did not fall under the Human Research Act (BASEC-Nr. Req-2021-00227).

### 2.2. Statistical analysis

Descriptive statistics were summarized as absolute frequencies and percentages for categorical variables and as means (± standard deviation) or medians (interquartile range) for continuous variables, depending on the normality of data distribution.

In both pilot study 1 and pilot study 2, each patient was categorized into either a “high ICEBERG (≥10)” or “low ICEBERG (<10)” group based on the observed median score of the included patients. Pearson chi-square analysis was used to compare the distribution by sex, while continuous variables were compared using two-sample *t*-tests. For non-normally distributed continuous variables, the Wilcoxon rank sum test was used to compare the two ICEBERG groups (≥10 vs. <10). Spearman rank correlation analysis was performed for the ICEBERG scores and all components of the CGA.

For pilot study 2, logistic regression analyses were used for four categorical outcomes as dependent variables: (1) need of one-on-one nursing care, (2) re-hospitalization within 30 days, (3) in-hospital mortality, and (4) discharge to a nursing home (among patients admitted from home). Odds ratios (ORs) with 95% confidence intervals (CI) were calculated, after adjusting for age and sex. All data analyses were 2-tailed, with *p* < 0.05 set as the criterion for statistical significance. Stata Special Edition statistical software version 16.0 (Stata Corp.) was used.

## 3. Results

### 3.1. Pilot 1

Pilot 1 included 129 geriatric patients summarized in Table 3. Mean age of patients in pilot 1 was 82.9 years (SD 6.7) and 83 (64%) were women. The median ICEBERG score was 10 (IQR 7, 16). Regarding construct validity of the ICEBERG tool with the CGA, performance in eight of the standardized tests of the CGA (all, except self-care index), was statistically significant worse in participants with an ICEBERG score of 10 and higher (Table 3). The individual correlation between the ICEBERG score and individual CGA components is presented in Supplementary Table 3.

**Table 3:** Findings pilot 1 (*N* = 129).

Variable	Overall *N* = 129	ICEBERG < 10 *N* = 60	ICEBERG ≥ 10 *N* = 69	*p*-value
Women (count, %)	83 (64.3%)	40 (66.7%)	43 (62.3%)	0.61
Age (Mean, SD)	82.9 (6.7)	81.2 (6.9)	84.4 (6.2)	0.05
SPI (Median, IQR)	30 (25.5–34)	30.5 (26.5–34)	29 (25–33)	0.37
Barthel index (Median, IQR)	50 (40–75)	60 (40–80)	50 (35–60)	<0.05
MMSE (Median, IQR)	25 (21–27)	26 (24–28)	22 (19–26)	<0.001
MNA (Median, IQR)	20.5 (19–22.5)	21.5 (20–23.5)	19 (17–22)	<0.001
Handgrip right-side (Median, IQR)	40 (31–51)	44 (35–53)	38 (28–49)	<0.05
Handgrip left-side (Median, IQR)	38.5 (27.5–50)	40 (31–52)	36 (23–47)	<0.05
SPPB (Median, IQR)	4 (2–6)	5 (3–8)	2 (1–5)	<0.05
Clock drawing test (Median, IQR)	5 (2–7)	6.5 (5–7)	3 (2–5)	<0.001
Frailty-fried (Median, IQR)	3 (2–4)	2 (2–3)	3 (3–4)	<0.001
Frailty-SHARE (Median, IQR)	2 (1–4)	1 (1–2)	3 (2–4)	<0.001

Self-care index (SPI, range 0–40), Mini Mental State Examination (MMSE, range 0–30), Barthel Index (range 0–100), Mini Nutritional Assessment (MNA, range 0–30), Handgrip right-side (kg), Handgrip left-side(kg), Short Physical Performance Battery (SPPB, range 0–12), Clock drawing test (range 0–7), Frailty score according to Fried (Frailty-Fried, range 0–5) and Frailty score according to SHARE (Frailty-SHARE, range 0–5).

Chi-square test was used to compare female distribution between two groups. *T*-test was used to compare the age difference between two groups. Wilcoxon rank sum tests are used to compare geriatric assessment outcomes between two groups.

### 3.2. Pilot 2

Pilot 2 included 288 patients summarized in Table 4. Mean age of pilot 2 participants was 81.5 (SD 7.0) years and 147 (51%) were women. Of the 288 enrolled patients, 87 were recruited at USZ, 68 at City Hospital Zurich-Waid, and 139 at City Hospital Zurich-Triemli.

**Table 4:** Findings pilot 2 (*N* = 288).

Variable (Median, IQR)	Overall *N* = 288	ICEBERG < 10 *N* = 129	ICEBERG ≥ 10 N = 159	*p*-value
Women (*n*, %)	147(51%)	67 (51.9%)	80 (50.3%)	0.78
Age (Mean, SD), years	81.5 (7.0)	80.0 (6.6)	82.7 (7.0)	<0.001
Time to finish the survey (Mean, SD), minutes	4.3 (1.3)	4.3 (1.3)	4.3 (1.4)	0.840
Length of stay, days (Median, IQR)	7 (3–10)	6 (3–8)	7 (4–11)	0.003
One-on-one nursing care need (*n*, %)	30 (10.4%)	7 (5.4%)	23 (14.5%)	0.013
Re-hospitalization within 1 month (*n*, %)	45 (15.6%)	19 (14.7%)	26 (16.4%)	0.71
In-hospital Mortality (*n*, %)	14 (4.9%)	4 (3.1%)	10 (6.3%)	0.22
Discharge to nursing home (*n*, %)	52 (18%)	10 (7.8%)	42 (26.4%)	<0.001
LEP nursing care, minutes (median, IQR)*	1,607 (892–3,138)	1,393 (743–2,390)	1838 (901–4,267)	0.041

* Only available at the time from two hospitals (USZ and Triemli); *T*-test was used to compare for age difference between two groups. The Wilcoxon rank sum test was used to compare length of stay and LEP nursing care. Chi-square test was used to compare female distribution, use of one-on-one nursing care need, re-hospitalization, mortalityand discharge to nursing home between two groups.

Regarding feasibility of the ICEBERG tool in the ER setting, participation rate was high at 97%, and the average time to complete the ICEBERG tool using direct patient information and available electronic health records in the ER was 4.3 min (SD 1.3). For the feasibility assessment, we also documented the source of information for each ICEBERG question used in pilot 2. Some of the questions were mostly based on information of the available electronic health records in the ER (i.e., age, social situation, fall as the reason for ER visit, polypharmacy), while others mostly depended on direct information provided by the patient or accompanying relatives (i.e., fall in the last 12 months, cognitive impairment, mood, delirium, ER or emergency GP visit in the last 6 months, help in activities of daily living (ADL) such as showering or bathing or general assistance at home by informal or professional care-givers, mobility, weight loss, and loss of appetite). For less than 10% of questions support by proxies or utilization of other sources (see Supplementary Table 1) as required.

In pilot 2, we also asked all 288 patients who had participated in the ER evaluation of the ICEBERG tool to provide feedback on the difficulty level for each question. Most questions were reported as “easy” from participants. Only the question about delirium (Q5) was reported being difficult for 6.3% of patients and could not be assessed in 3.5% of patients (see Supplementary Table 2).

**Figure 2:**
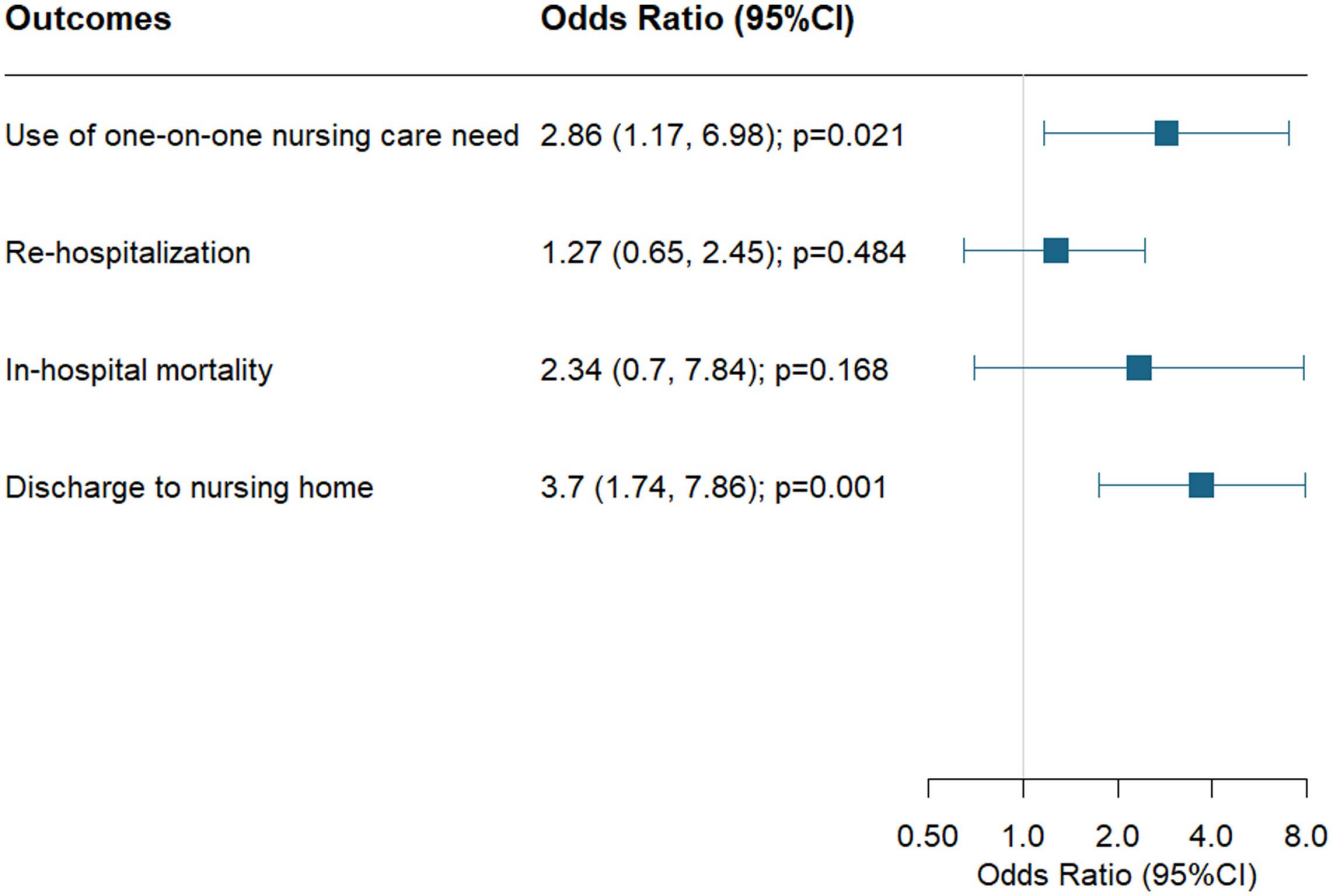
Association of higher ICEBERG scores (≥10) and outcomes in acute care logistic regression models are adjusted by age and sex.

Regarding criterion validity of clinically relevant outcome measures in pilot 2, patients above the median ICEBERG score (≥10) had a longer average LOS in acute care (median 7 days with IQR 4, 11 vs. median 6 days with IQR 3, 8) and required more nursing care time (median 1,838 min; IQR 901, 4,267 vs.) compared to patients below an ICEBERG score of 10 (median 1,393 min; IQR 743, 2,390). Further, patients with an ICEBERG score ≥ 10 had 2.9-fold higher odds of requiring one-on-one nursing care (OR = 2.86, 95% CI 1.17–6.98), and 3.7-fold higher odds of being discharged to a nursing home (OR = 3.70, 95%CI 1.74–7.85). Patients with an ICEBERG score ≥ 10 also had a higher in-hospital mortality and were re-hospitalized more often, however this did not reach statistical significance. Results of pilot 2 are summarized in Table 4 and Figure 2.

## 4. Discussion

In this paper we summarize the development and validation of ICEBERG as a novel and practical tool for early identification of geriatric consultation need among older patients admitted to acute care via the ER. The ICEBERG tool was designed to be comprehensive across nine geriatric domains that pose older patients at increased risk of adverse outcomes in acute care. For this, pilot 1 supports construct validity of the ICEBERG tool with regard to the CGA, while pilot 2 supports feasibility of the tool in the ER setting with regard to patients’ acceptance to participate, ability to respond to the ICEBERG questions, and with regard to the time needed required to perform ICEBERG in the ER. Further, pilot 2 supports criterion validity of ICEBERG with regard to relevant clinical outcome indicators, including length of stay and nursing care time, as well as the odds of one-on-one care needs and the odds of discharge to a nursing home.

Early identification of underlying geriatric risk factors in older patients admitted to the ER is gaining clinical importance due to the expected doubling of older adults by 2050 in both Europe and the US (1). The ICEBERG tool may help identify an underlying geriatric risk profile in this growing patient group in about four minutes in the ER, with a high acceptance by the patient and demonstrated feasibility.

Compared with the gold standard CGA, the ICEBERG showed construct validity where participants who scored ICEBERG at 10 or more performed significantly worse in 8 of 9 standardized tests of the CGA, including cognitive function, mobility, nutritional state, mental health, muscle strength, ADL, and frailty.

In comparison, the most widely used ISAR screening tool does not capture delirium risk, fall risk/gait disturbances, and malnutrition (27). Similarly, other existing tools do not cover geriatric domains in the same comprehensiveness as ICEBERG (7–12).

Regarding criterion validity, our prospective pilot 2 suggests that ICEBERG predicts longer length of stay in acute care, higher amount of nursing care time, higher odds for one-on-one nursing care, and higher odds of being discharged to a nursing home. Additionally, ICEBERG may predict higher in-hospital mortality and increased risk for hospital re-admission within 30 days, although this could not be confirmed in our study and needs validation in a larger study. Regarding other ER screening tools, ISAR was found to predict re-hospitalization (28), and Silver Code to predict 1-year mortality (8).

The current study has several strengths. First, the ICEBERG tool was developed based on a detailed review of previously published screening tools and the input of geriatric experts. Second, our study addressed and demonstrated the feasibility of the ICEBERG tool in the ER setting, and two validation studies support construct validity with the CGA, and demonstrated criterion validity for key clinical outcome indicators.

The current study also has limitations. First, both validation studies had a pilot format, and our findings warrant further investigation in a larger sample of ER patients. Additionally, we missed significance for two investigated clinical outcome indicators, in-hospital mortality and 30-day readmission rate, likely due to the small sample size of the pilot 2.

In summary, we describe the development, feasibility, and pilot validity of ICEBERG, a novel and practical assessment tool for the ER setting. Based on our findings ICEBERG may help identify older patients early in need of geriatric consultation. Given the pilot format of our validation efforts, these findings require confirmation in a larger study.

## Data availability statement

The raw data supporting the conclusions of this article will be made available by the authors, without undue reservation.

## Ethics statement

The studies involving humans were approved by Ethics Committee of the Canton of Zurich, Switzerland. The studies were conducted in accordance with the local legislation and institutional requirements. The Ethics Committee/Institutional Review board waived the requirement of written informed consent for participation from the participants or the participants' legal guardians/next of kin because the Cantonal Ethics Committee of Zurich declared that authorization from the Ethics Committee was not required as this project did not fall under the Swiss Human Research Act (BASEC-Nr. Req-2021-00227).

## Author contributions

HB-F had full access to all the data in the study and takes responsibility for the integrity of the data and the accuracy of the data analysis. HB-F conceived and designed the study, with input by MG, D-HT, PL, PS, UC, DK, KG, and GF. All authors acquired, analyzed, and interpreted the data. HB-F, MG, and D-HT drafted the manuscript, with input by all authors. All authors critically revised the manuscript for important intellectual content. HB-F and D-HT statistically analyzed the study. HB-F obtained the funding. All authors contributed to the article and approved the submitted version.
