# Supplementary material for: Validation of the ICEBERG emergency room screening tool for early identification of older patients with geriatric consultation needs
Source: Front Med (Lausanne). 2023 Sep 27;10:1240082. doi: 10.3389/fmed.2023.1240082 (PMC10565002; doi:10.3389/fmed.2023.1240082)
Supplement: Supplementary file 1 [file Table_1.DOCX]

**Supplementary File for**

**Validation of the ICEBERG emergency room screening tool for early identification of older patients with geriatric consultation needs**

Heike A Bischoff-Ferrari ^a,b,c^, *Michael Gagesch ^a,c^, Dai-Hua Tsai ^a^, Clara Richter ^a^, Patricia Lanz ^b^, Patrick Sidler ^d^, Uenal Can ^d^, Dagmar I Keller ^e^, Markus Minder ^f^, Bettina von Rickenbach ^f^, Ali Yirdim-Aman ^g^, Katharina Geiling ^a,c^, and Gregor Freystaetter ^a,c^

**Supplementary Table 1:** Sources for ICEBERG data in pilot 2 (N=288; %)

| Questions | Electronic Health Records | Patient | Proxy | Other |
| --- | --- | --- | --- | --- |
| Q1. Social situation | 121 (42.0%) | 149 (51.7%) | 16 (5.6%) | 2 (0.7%) |
| Q2. Age | 286 (99.3%) | 2 (0.7%) | --- | --- |
| Q3a. Falls in last 12m | 26 (9.1%) | 236 (82.8%) | 23 (8.1%) | --- |
| Q3b. Fall as reason for ER visit | 227 (78.8%) | 60 (20.8%) | 1(0.4%) | --- |
| Q4a. Cognitive disorder | 33 (11.5%) | 224 (78.1%) | 30 (10.5%) | --- |
| Q4b. Mood | 1 (0.4%) | 261 (92.6%) | 19 (6.7%) | 1 (0.4%) |
| Q5. Delirium | --- | 276 (98.9%) | 1 (0.4%) | 2 (0.7%) |
| Q6a. ER or Emergency GP visit in last 6m | 82 (28.6%) | 187 (65.2%) | 17 (5.9%) | 1 (0.4%) |
| Q6b. Hospitalization in last 6m | 98 (34.4%) | 173 (60.7%) | 13 (4.6%) | 1 (0.4%) |
| Q7. Polypharmacy | 258 (89.6%) | 29 (10.1%) | --- | 1 (0.4%) |
| Q8a. Help for ADL at home | 11 (3.9%) | 245 (86.0%) | 28 (9.8%) | 1 (0.4%) |
| Q8b. Mobility | 1 (0.4%) | 264 (94.0%) | 16 (5.6%) | 3 (1.1%) |
| Q9a. Weight loss | 8 (2.8%) | 252 (89.1%) | 22 (7.8%) | 1 (0.4%) |
| Q9b. Appetite loss | 7 (2.5%) | 252 (89.1%) | 22 (7.8%) | 2 (0.7%) |

**Supplementary Table 2:** Difficulty level of each ICEBERG question for patients in the ER in pilot 2 (N=288; %)

|  | Difficult | Easy | missing |
| --- | --- | --- | --- |
| Q1. Social situation | 0 | 288 (100%) | 0 |
| Q2. Age | 0 | 288 (100%) | 0 |
| Q3a. Falls in last 12m | 2 (0.7%) | 283 (98.3%) | 3 (1.0%) |
| Q3b. Fall as reason for ER visit | 1 (0.3%) | 287 (99.7%) | 0 |
| Q4a. Cognitive disorder | 0 | 287 (99.7%) | 1 (0.3%) |
| Q4b. Mood | 1 (0.3%) | 280 (97.2%) | 7 (2.4%) |
| Q5. Delirium* | 18 (6.3%) | 260 (90.3%) | 10 (3.5%) |
| Q6a. ER or Emergency GP visit in last 6m | 0 | 286 (99.3%) | 2 (0.7%) |
| Q6b. Hospitalization in last 6m | 2 (0.7%) | 283 (98.3%) | 3 (1.0%) |
| Q7. Polypharmacy | 0 | 287 (99.7%) | 1 (0.3%) |
| Q8a. Help for ADL at home | 0 | 283 (98.3%) | 4 (1.4%) |
| Q8b. Mobility | 0 | 283 (98.3%) | 4 (1.4%) |
| Q9a. Weight loss | 0 | 282 (97.9%) | 6 (2.1%) |
| Q9b. Appetite loss | 1 (0.3%) | 281 (97.6) | 6 (2.1%) |

**For delirium 3.5% patients (n=10) had different reasons and situations impeding to answer this question. This included medical factors, e.g. patient was nauseous, could not answer, did not want to, other medical issues, daughter helped to answer, or had a language barrier.*

**Supplementary Table 3.** Spearman’s correlation coefficient between ICEBERG score and the variables comprehensive geriatric assessment in Pilot 1 (N=129).

| Variables | ICEBERG | Age | SPI | MMSE | BARTHEL-INDEX | MNA | HGS  (right hand) | HGS  (left hand) | SPPB | CDT | FRIED | SHARE |
| --- | --- | --- | --- | --- | --- | --- | --- | --- | --- | --- | --- | --- |
| ICEBERG | 1.00 |  |  |  |  |  |  |  |  |  |  |  |
| Age | 0.27^**^ | 1.00 |  |  |  |  |  |  |  |  |  |  |
| SPI | -0.17 | -0.06 | 1.00 |  |  |  |  |  |  |  |  |  |
| MMSE | -0.37^***^ | -0.10 | 0.34^***^ | 1.00 |  |  |  |  |  |  |  |  |
| BARTHEL-I. | -0.27^**^ | -0.04 | 0.60^***^ | 0.34^***^ | 1.00 |  |  |  |  |  |  |  |
| MNA | -0.40^***^ | -0.07 | 0.26^**^ | 0.36^***^ | 0.28^**^ | 1.00 |  |  |  |  |  |  |
| HGS (right) | -0.22^*^ | -0.37^***^ | 0.33^***^ | 0.29^**^ | 0.28^**^ | 0.24^*^ | 1.00 |  |  |  |  |  |
| HGS (left) | -0.20^*^ | -0.47^***^ | 0.32^***^ | 0.34^***^ | 0.31^**^ | 0.23^*^ | 0.69^***^ | 1.00 |  |  |  |  |
| SPPB | -0.32^**^ | -0.23^*^ | 0.57^***^ | 0.23^*^ | 0.61^***^ | 0.28^**^ | 0.31^**^ | 0.41^***^ | 1.00 |  |  |  |
| CDT | -0.42^***^ | -0.17 | 0.28^**^ | 0.47^***^ | 0.26^*^ | 0.40^***^ | 0.24^*^ | 0.18 | 0.37^**^ | 1.00 |  |  |
| FRIED | 0.40^***^ | 0.22^*^ | -0.27^**^ | -0.24^*^ | -0.29^**^ | -0.25^**^ | -0.50^***^ | -0.46^***^ | -0.52^***^ | -0.30^**^ | 1.00 |  |
| SHARE | 0.49^***^ | 0.20^*^ | -0.33^***^ | -0.35^***^ | -0.30^**^ | -0.33^***^ | -0.37^***^ | -0.29^**^ | -0.51^***^ | -0.37^***^ | 0.72^***^ | 1.00 |

** p < 0.05, ** p < 0.01, *** p < 0.001; HGS, Handgrip Strength; SPI, Self-Care Index; SPPB, Short Physical Performance Battery; MNA, Mini Nutritional Assessment; MMSE, Mini Mental Status Examination; CDT, Clock Drawing Test.*
